# Re. van den Berg et al, Deep learning for automated contouring of neurovascular structures on magnetic resonance imaging for prostate cancer patients

**DOI:** 10.1016/j.phro.2023.100513

**Published:** 2023-11-17

**Authors:** Nikolaos Grivas, Inge Cox, Thierry Boellaard, Henk van der Poel

**Affiliations:** a Department of Surgical Oncology (Urology), Netherlands Cancer Institute, 1066 CX Amsterdam, The Netherlands; b Department of Laparoscopy and Endourology, Central Urology, Lefkos Stavros Clinic, Athens, Greece; c Department of Radiology, Netherlands Cancer Institute, 1066 CX Amsterdam, The Netherlands

In the era of personalized medicine, magnetic resonance-guided radiotherapy (MRgRT) is a new technique aiming to reduce radiation-induced toxicity while improving oncologic outcomes by facilitating dose-escalation [1]. Recent series have shown that neurovascular bundle sparing with the application of MRgRT, can preserve erectile function in almost half of the patients [2]. Technical aspects such as automatically contouring of neurovascular structures on prostate Magnetic Resonance Imaging with artificial intelligence systems can improve the results of the technique, improve workflow and limit interrater disagreement. In this field of very limited data, van den Berg et al. [3] should be congratulated for comparing the efficacy of two auto-contouring, deep learning networks, nnU-Net and DeepMedic. The nnU-Net showed excellent contouring performance allowing high interrater agreement between radiation oncologists [3]. In our previous series [4] we also used a semi-automated system (macro in imageJ) to measure the fascia surface and fascia thickness in preoperative Magnetic Resonance Imaging. In our cohort of 106 patients, fascia thickness and fascia preservation were independent predictors of post-prostatectomy erectile dysfunction [4]. The mid-prostate level had the highest intraclass correlation coefficient among observers [4]. An interesting observation is that our measured median fascia thickness was 3.8 mm while in the authors study [3] the average surface distance was 0.7–0.9 mm. Moreover, earlier data suggest that lateral and more ventral regions of the fascia surrounding the prostate do contribute to erectile function when preserved during prostatectomy [5].

These differences show that there can be variations in different model’s performance. Therefore, each model should be externally validated before routine clinical use. Application and evolution of artificial intelligence systems and incorporation of the results in 3D systems can also assist magnetic resonance imaging-guided nerve sparing surgery and improve erectile function preservation after radical prostatectomy. The research field is open and better training is essential both for automatic models as well as for radiation oncologists.

## Declaration of Competing Interest

The authors declare that they have no known competing financial interests or personal relationships that could have appeared to influence the work reported in this paper.
